# Reactive oxygen species–mediated switching expression of MMP-3 in stromal fibroblasts and cancer cells during prostate cancer progression

**DOI:** 10.1038/s41598-017-08835-9

**Published:** 2017-08-22

**Authors:** Chia-Ling Hsieh, Che-Ming Liu, Hsin-An Chen, Shun-Tai Yang, Katsumi Shigemura, Koichi Kitagawa, Fukashi Yamamichi, Masato Fujisawa, Yun-Ru Liu, Wei-Hua Lee, Kuan-Chou Chen, Chia-Ning Shen, Cheng-Chieh Lin, Leland W. K. Chung, Shian-Ying Sung

**Affiliations:** 1 The Ph.D. Program for Translational Medicine, College of Medical Science and Technology, Taipei Medical University, Taipei, Taiwan; 2 The Ph.D. Program for Cancer Biology and Drug Discovery, China Medical University and Academia Sinica, Taichung, Taiwan; 3 Department of Surgery, School of Medicine, College of Medicine, Taipei Medical University, Taipei, Taiwan; 4 Division of General Surgery, Department of Surgery, Shuang Ho Hospital, Taipei Medical University, New Taipei City, Taiwan; 5 Graduate Institute of Clinical Medicine, College of Medicine, Taipei Medical University, Taipei, Taiwan; 6 Department of Neurosurgery, Shuang Ho Hospital, Taipei Medical University Taiwan, Taipei, Taiwan; 7 Department of Urology, Kobe University Hospital, Kobe, Japan; 8 Department of International Health, Kobe University Graduate School of Health Science, Kobe, Japan; 9 Department of Urology, Hyogo Prefectural Amagasaki Hospital, Amagasaki, Hyogo Prefecture 660-0828 Japan; 10 Joint Biobank, Office of Human Research, Taipei Medical University, Taipei, Taiwan; 11 Department of Pathology, Shuang Ho Hospital, Taipei Medical University, New Taipei City, Taiwan; 12 Department of Urology, Shuang Ho Hospital, Taipei Medical University, New Taipei City, Taiwan; 13 Department of Urology, College of Medicine, Taipei Medical University, Taipei, Taiwan; 14 Genomic Research Center, Academia Sinica, Taipei, Taiwan; 15 Uro-oncology Research Program, Samuel Oschin Comprehensive Cancer Institute, Cedars-Sinai Medical Center, Los Angeles, CA 90048 USA; 16 Joint Clinical Research Center, Office of Human Research, Taipei Medical University, Taipei, Taiwan

## Abstract

Studies on the aberrant control of extracellular matrices (ECMs) have mainly focused on the role of malignant cells but less on that of stromal fibroblasts during cancer development. Herein, by using paired normal and prostate cancer-associated stromal fibroblasts (CAFs) derived from a coculture cell model and clinical patient samples, we demonstrated that although CAFs promoted prostate cancer growth, matrix metalloproteinase-3 (MMP-3) was lower in CAFs but elevated in prostate cancer cells relative to their normal counterparts. Furthermore, hydrogen peroxide was characterized as the central modulator for altered MMP-3 expression in prostate cancer cells and CAFs, but through different regulatory mechanisms. Treatment of CAFs but not prostate cancer cells with hydrogen peroxide directly inhibited *mmp-3* promoter activity with concomitant nuclear translocation of nuclear factor-κB (NF-κB), indicating that NF-κB is the downstream pathway for the transcriptional repression of MMP-3 in CAFs. Hydrogen peroxide reduced thrombospondin 2 (an MMP-3 suppressor) expression in prostate cancer cells by upregulating microRNA-128. To the best of our knowledge, this is the first study to demonstrate the crucial role of reactive oxygen species in the switching expression of MMP-3 in stromal fibroblasts and prostate cancer cells during tumor progression, clarifying how the tumor microenvironment modulates ECM homeostasis control.

## Introduction

Cancer progression is a complex process involving local invasion, micrometastasis, and intravasation. The invasive capacity of cancer cells is dependent on their ability to cleave the extracellular matrix (ECM) and basement membranes surrounding epithelial cells as well as to remodel ECM components. Matrix metalloproteinases (MMPs), a well-studied protein family, are responsible for the dynamic regulation of environmental shedding before cancer cell migration and invasion (micrometastasis)^1, 2^. Consequently, there is considerable interest in identifying factors influential in MMP signaling and the regulation of environmental changes required for cancer invasion. In addition, developing pharmacological inhibitors of MMPs may provide clinical benefits through the suppression of local dissemination and metastatic spread^3, 4^.

Studies of cancer gene changes have revealed MMP expressions in cancer cells that play crucial roles in cancer progression^5–7^; however, the regulation of MMP expression in cancer-associated fibroblasts (CAFs) has not been fully explored^6^. The ability of tumor cells to move through tissues involves both remodeling of the ECM and enhancement of cell mobility. Each step requires reciprocal communication, involving cell–cell, cell–insoluble ECMs, and cell–soluble factor-mediated signaling processes, between tumor cells and host stroma^8, 9^. During cancer micrometastasis, changes in ECM factors lead to the generation of a special trail through the localizing and clustering of MMP activities. Consequently, different cells in the tumor microenvironment may have different regulatory mechanisms to satisfy the requirements for cancer cell movement; for example, the release of chemoattractants and ECM remodeling require reactive stromal cell activation^10–13^.

Stromelysin 1 (MMP-3) and 2 (MMP-10) exhibit increased expression in various tumors and thus influence cancer initiation and the neoplastic risk^5, 7, 14, 15^. Expression of the Rac1 isoform Rac1b by cancer cells induces MMP-3 expression^15^. Furthermore, MMP-3 overexpression occurs through mediation by reactive oxygen species (ROS)^15^. Therefore, MMP-3 and -10 expressions are mostly regulated at the gene transcriptional level by environmental stimuli, including ROS, growth factors, cytokines, and tumor factors^15–17^. In addition, single-nucleotide polymorphism-based studies have demonstrated that promoter polymorphisms alter stromelysin expression levels, such as −1171 5 A/5 A in MMP-3^18, 19^. However, most studies have investigated the relationship between MMP-3 and cancer progression with a focus on cancer cells and not on stromal fibroblasts, which are the major cells expressing MMPs. Elucidating the homeoregulation of stromelysin between cancer cells and host cells in the tumor milieu would provide a better understanding of the critical role of reciprocal stromal–epithelial interactions in controlling cancer progression.

In the present study, we focused on profiling the expression pattern of ECM remodeling-related genes associated with prostate cancer development in paired CAFs and normal fibroblasts derived from a coculture cell model and clinical patient samples. Although CAFs exhibited higher capacity to promote prostate cancer tumor formation, these cells expressed lower levels of MMP-3 than did normal fibroblasts. By contrast, prostate cancer cells exhibited increased MMP-3 expression, which was correlated with tumor grade. Moreover, we provide the first evidence that hydrogen peroxide serves as a central mediator in regulating MMP-3 expression, with opposite results in the microenvironments of fibroblasts and prostate cancer cells, through the direct inhibition of *mmp-3* promoter activity via nuclear factor-κB (NF-κB) signaling pathway in CAFs and downregulation of thrombospondin 2, an MMP-3 suppressor in prostate cancer cells through microRNA (miRNA) regulation.

## Results

### CAFs promote prostate cancer growth

**Figure 1:**
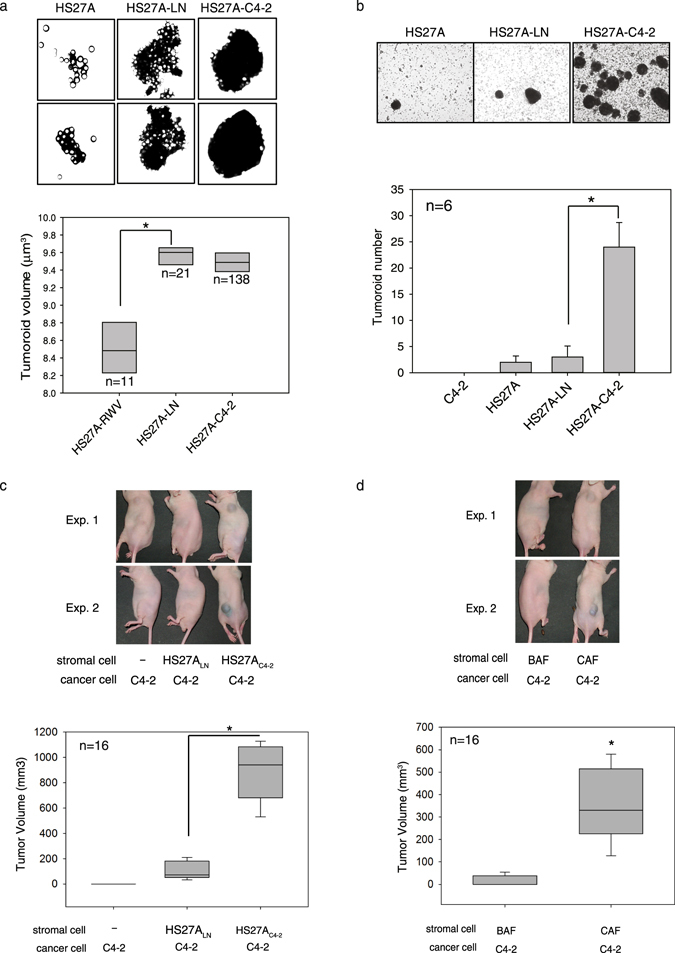
Interactions of stromal fibroblasts and prostate cancer. Coculture of HS27A bone stromal fibroblasts with prostate cancer LNCaP (HS27A-LN) and C4-2 (HS27A-C4-2) cells as tumoroids or alone (HS27A) under 3D-RWV culture conditions (**a**,**b**). Microscopic images of cultures were taken on day 14, and the size (**a**) and number of tumoroids were calculated. Each condition was performed in duplicate vessels for three independent experiments. A representative image of each group is shown at the top. Quantitative data are represented as a box–whisker plot of triplicate experiments. Tumor-promoting effect of cancer-associated fibroblasts (CAFs) in a xenograph mouse model (**c**,**d**). Nude mice were subcutaneously implanted with C4-2 prostate cancer cells, either alone (C4-2) or mixed with HS27A pre-cultured with LNCaP (HS27A_LN_) or C4-2 (HS27A_C4-2_) (**c**), or primary cultured prostate fibroblasts derived from benign/normal prostate (BAF) or prostate cancer (CAF) tissues of the same patients (**d**). Eight mice were used in each group and independently conducted twice. Tumor growth was monitored weekly, and the results are presented as representative images of two independent experiments (top) and a box–whisker plot of median tumor volumes (bottom) of each group (*n* = 16 per group; two independent experiments pooled) at the end of the experiment (8 weeks after cell inoculation). Boxes (**a**–**d**) encompass the 25th to 75th percentiles, and the whiskers represent the 10%–90% range of observations. Lines within boxes represent median values. *p < 0.001.

Prostate cancer cells preferentially metastasize to bones. Therefore, we attempted to determine whether CAFs result in prostate cancer progression at both primary and metastatic sites. Because tissue resources of prostate cancer bone metastases are rare and difficult to collect, we coinoculated prostate cancer cells and HS27A bone stromal cells under three-dimensional (3D) conditions in the rotary cell culture system (RCCS), an experimental model widely used for establishing matched pairs of normal and cancer-associated stromal cell lines that mimic tumor–stroma coevolution^20, 21^. We first noted that the coculture of HS27A cells with either LNCaP (HS27A-LN) or C4-2 cells (HS27A-C4-2) formed larger-sized ball-like cellular aggregates (chimeric tumoroids), which were more than 10 times larger than HS27A monoculture aggregates (HS27A-RWV) (Fig. 1a). Furthermore, an increased number of tumoroids was found when prostate cancer cells were cocultured with HS27A cells, particularly HS27A-C4-2 formed a 12-fold higher number of aggregates than did HS27A-RWV (Fig. 1b). By contrast, growing C4-2 prostate cancer cells under RCCS conditions (C4-2-RWV) did not show grossly visible tumoroids, suggesting strong cell–cell interactions between prostate cancer cells and bone stromal fibroblasts. Immunohistochemical analysis showed intense staining of the luminal epithelial marker cytokeratin 8/18 in aggregates harvested from HS27A-LNCaP and HS27A-C4-2 coculture groups but not in aggregates of the HS27A-RWV monoculture (Supplemental Fig. s1, CK8/18), whereas the positive staining of the fibroblast marker vimentin showed ubiquitous distribution with homogeneous intensity throughout the HS27A-RWV section and a spatial expression pattern in the HS27A-LNCaP and HS27A-C4-2 groups (Supplemental Fig. s1, Vimentin). This result confirmed that chimeric tumoroids comprised prostate cancer cells and HS27A cells under these coculture conditions. We isolated HS27A cells from monoculture aggregates and each chimeric tumoroid. The resultant HS27A derivatives were designated as genetically relevant normal stromal fibroblast (HS27A_RWV_) and prostate cancer-associated stromal fibroblast cell lines (HS27A_LN_ and HS27A_C4-2_) for additional studies.

To validate whether CAFs promote prostate cancer growth *in vivo*, we performed subcutaneous coinoculation of prostate cancer cells and stromal fibroblasts in mice. We observed tumor formation in the group of mice transplanted with the HS27A_LN_/C4-2 and HS27A_C4-2_/C4-2 mixtures but not with C4-2 alone. Inoculation of the HS27A_C4-2_/C4-2 mixture formed significantly larger tumors than did HS27A_LN_/C4-2, with average sizes of 893.4 ± 231.7 and 107.4 ± 72.2 mm^3^ (two-tailed Student’s *t*-test, *p* ≤ 0.001), respectively (Fig. 1c). In a parallel study, HS27A_C4-2_ and HS27A_LN_ were inoculated alone, and the result confirmed the nontumorigenic property of HS27A derivatives. Given that C4-2 is an aggressive subline of LNCaP, the tumor-promoting activity of cancer-associated HS27A is correlated with the aggressiveness of prostate cancer cells with which they interacted. This protumorigenic activity of CAFs shown by the bone stromal cell lines HS27A_C4-2_ and HS27A_C4-2_ was validated using the characterized paired prostate CAFs and benign-associated fibroblasts (BAFs) that were previously established from clinical specimens^21^. The tumorigenesis analysis of the xenograft mouse study indicated an approximate 20-fold increase in tumor volumes in the CAF/C4-2 coinoculation group (362.4 ± 75.0 mm^3^) compared with the BAF/C4-2 coinoculation group (15.4 ± 10.6 mm^3^) (Fig. 1d). These results demonstrate that CAFs exhibited a higher ability to accelerate prostate cancer growth than did BAFs.

### MMP-3 expression is lower in prostate cancer-associated stromal fibroblasts but higher in cancer epithelial cells

**Figure 2:**
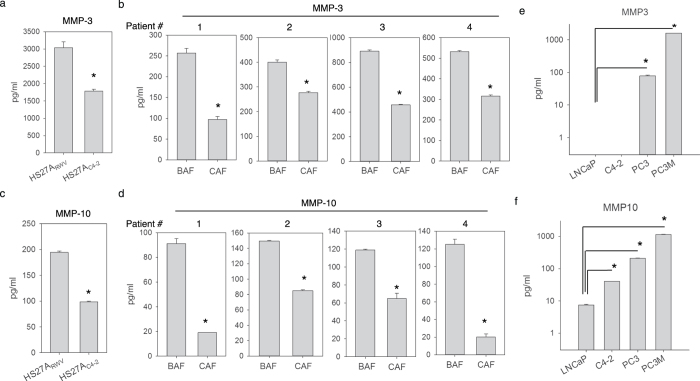
Detection of stromelysin expression in paired normal and cancer-associated stromal fibroblasts and prostate cancer cell lines. Conditioned media obtained from the normal HS27A_RWV_ and prostate cancer-associated HS27A_C4-2_ bone stromal fibroblasts (**a**,**c**), four paired benign- and cancer-associated prostate fibroblasts (**b**,**d**), and a serial of prostate cancer cell lines (**e**,**f**) were analyzed through an ELISA for quantitative detection of stromelysin 1 (MMP-3) (**a**,**b**,**e**) and stromelysin 2 (MMP-10) (**c**,**d**,**f**) protein expressions. Assays were performed in three independent experiments in triplicates. Data are presented as the mean ± SD of triplicate determinations of one representative experiment. *p < 0.001.

MMP expression levels have previously been correlated with cancer malignant progression^5, 22, 23^. However, the cellular distributions of MMPs have not been fully illustrated. To determine whether the expression patterns of stromal cell-derived MMPs, such as stromelysin, are altered during prostate cancer progression, we performed an ECM microarray analysis (Supplemental Fig. s3a) to compare the gene expression levels of a panel of extracellular components between HS27A_C4-2_ and HS27A_RWV_ and between CAFs and BAFs. The results were verified using real-time quantitative polymerase chain reaction analysis, which revealed significantly decreased expressions of Selenbp1, IGFBP7, IL13RA2, and stromelysin 1 (MMP-3) genes in both HS27A_C4-2_ cells and CAFs compared with their corresponding normal compartments of HS27A_RWV_ and BAFs, respectively (Supplemental Fig. s3b). A pathway-focused antibody array analysis demonstrated a similar differential expression of MMP-3 by HS27A_C4-2_ and CAFs at the protein level (Supplemental Fig. s3c). Among the 11 tested MMPs and the tissue inhibitor of metalloproteinases (TIMPs), MMP-3 and MMP-10 (stromelysin 2) were significantly lower in HS27A_C4-2_ and all four patients’ CAFs. Zymographic analysis of stromelysin activity using casein gel confirmed the decreased stromelysin activity in the conditioned medium (CM) of CAF relative to BAF (Supplemental Fig. s3d). To quantitatively determine the concentrations of released MMP-3 and MMP-10 in the CM, the CM collected from these cells was subjected to enzyme-linked immunosorbent assay (ELISA). The average MMP-3 level was 1.7-fold lower in HS27A_C4-2_ than in HS27A_RWV_ (Fig. 2a). Similarly, we observed a 1.4–2.5-fold decrease in MMP-3 in the CM of CAFs compared with paired BAFs (Fig. 2b). Similarly, MMP-10 was 1.9-fold lower in HS27A_C4–2_ (Fig. 2c) and 1.8–6.2-fold lower in four paired CAFs (Fig. 2d).

To determine whether prostate cancer cells reduce stromelysin expression during cancer progression similar to stromal fibroblasts, we collected the CM of the androgen-responsive LNCaP lineage (LNCaP and C4-2) and androgen-insensitive PC3 lineage (PC3 and PC3M) prostate cancer cell lines and measured MMP-3 and -10 concentrations. Unexpectedly, sMMP-3 was undetectable in LNCaP lineage cell lines, and a significantly (approximately 20-fold) higher concentration of MMP-3 was found in highly metastatic PC3M cells (1594 ± 0.6 pg/ml) than in their parental PC3 cells (78.5 ± 4.2 pg/ml) (Fig. 2e). Similarly, average concentrations of MMP-10 were approximately 5-fold higher in both C4-2 (40.0 ± 0.5 pg/ml) and PC3M (1140.0 ± 19.1 pg/ml) cell lines than in their lineage-related and less aggressive LNCaP (7.5 ± 0.3 pg/ml) and PC3 (209.0 ± 3.3 pg/ml) cells, respectively (Fig. 2f). These results indicate a correlation between elevated stromelysin expressions and aggressive progression of prostate cancer cells. In addition, treatment of C4-2 cells that did not express sMMP-3 with the CM harvested from highly expressing PC3 and PC3M cells significantly increased cell motility with no obvious effect on cell proliferation (Supplemental Fig. s2). A similar result was also observed in exogenous MMP-3 treatment group, suggesting that stromelysins (at least MMP-3) are critical for determining the invasiveness of prostate cancer.

**Figure 3:**
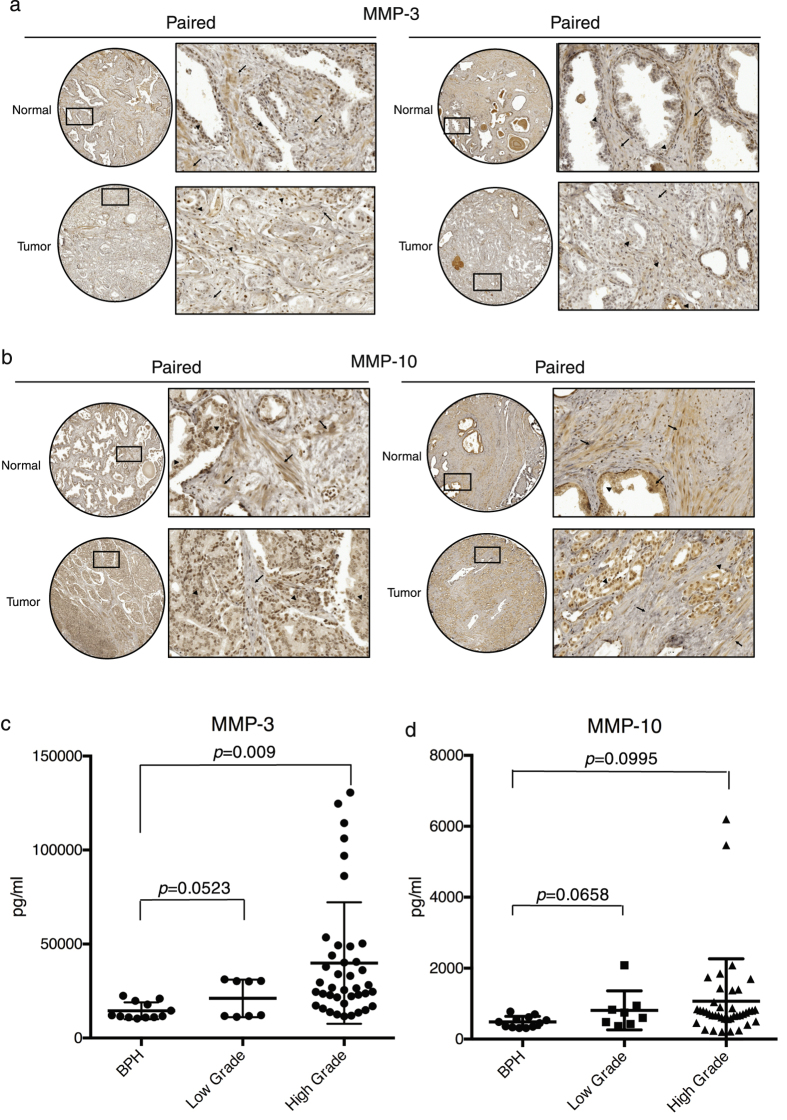
Detection of MMP-3 and -10 expression in clinical specimens of prostate cancer patients. Representative digital images of IHC staining of MMP-3 (**a**) and MMP-10 (**b**) in paired prostate tumor and normal prostate tissues from two individual patients in sections of a human prostate tissue microarray. Left, overview image of the tissue core; right, enlargement of the indicated area. The arrow and arrowhead respectively indicate positive staining in stromal and epithelial cells. Serum samples from benign prostatic hyperplasia (BPH) patients (*n* = 12) and prostate cancer patients with a Gleason score ≤ 7 (low grade, *n* = 8) or ≥8 (high grade, *n* = 40) were tested for MMP-3 through an ELISA (**c**). Statistical analysis was performed using unpaired Student’s *t*-tests.

Consistent with the cell lines, in nine matched pairs of prostate tumor and normal prostate samples, we found stronger positive immunostaining of MMP-3 (eight of nine cases, 89%) and MMP-10 (six of nine cases, 67%) in the stromal components of normal prostate tissues than in those of paired malignant tumor tissues (Fig. 3a,b). In epithelial components, MMP-3 staining was intensively positive in tumor cells and higher than that in normal glands. By contrast, no obvious differences in MMP-10 expression levels were found between normal and cancer epithelia. We next examined whether the serum levels of MMP-3 contributed by both stromal and tumor cells were associated with disease progression. ELISA indicated a trend of increased serum MMP-3 level with malignant cancer progression (Fig. 3c). The MMP-3 serum level in prostate cancer patients with high-grade (Gleason score ≥ 8, *n* = 40) tumors was 39,883 ± 32,333 pg/ml, which was significantly higher than that in patients with benign prostatic hyperplasia (BPH) (14,494 ± 4,484 pg/ml, *n* = 12). Similarly, patients with low-grade tumors (Gleason score ≤ 7) had a higher serum MMP-3 level (21,096 ± 10,023 pg/ml, *n* = 8) than did patients with BPH, but the difference was in the margin of significance (*p* = 0.0523). By contrast, there was no statistical significance in the serum MMP-10 concentration (Fig. 3d) between BPH (484.7 ± 157.7 pg/ml) and prostate cancer patients with either low-grade tumors (810.7 ± 552.2 pg/ml) or high-grade tumors (1069.3 ± 1197.3 pg/ml). Collectively, these clinical observations indicate that MMP-3 alone—and not both MMP-3 and MMP-10—exhibits opposite expression patterns in stromal fibroblasts and prostate cancer cells and an overall increase in MMP-3 in patient sera. Large variations in serum MMP-3 levels observed in the cancer patient group in the present study are attributable to the increased number of malignant epithelial cells, which potentially compromised the decreased expression of MMP-3 in CAFs at different levels.

### Hydrogen peroxide regulates MMP-3 expression in prostate cancer cells and stromal fibroblasts in a reverse manner

To identify candidate mediators that regulate genetic changes in stromal cells during prostate cancer progression, we performed Ingenuity Pathway Analysis (IPA, http://www.ingenuity.com ) on transcripts differentially expressed between HS27A_RWV_ and HS27A_C4-2_ and between CAFs and BAFs. Transforming growth factor beta-1 (TGFB1) and hydrogen peroxide were found to be central mediators in the altered molecular network seen in both HS27A_C4-2_ and CAFs compared with their corresponding controls (Supplemental Fig. s3b and s4a), suggesting a regulatory role of TGFB1 and/or hydrogen peroxide in stromal gene expression, including that of MMP-3.

**Figure 4:**
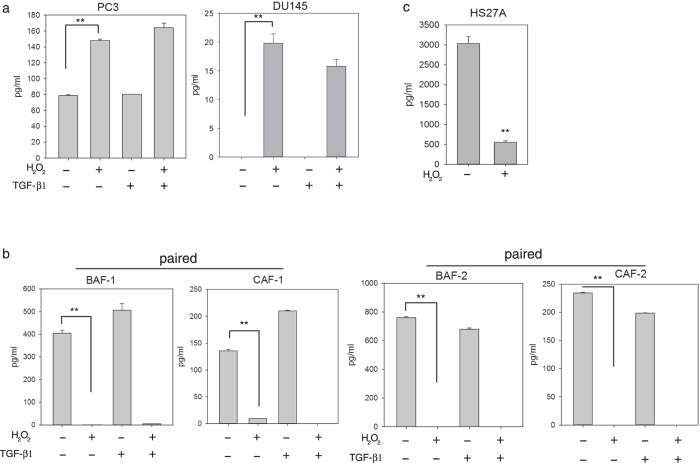
Effect of hydrogen peroxide and transforming growth factor (TGF)-β1 on MMP-3 protein expression in prostate cancer and stromal cells. An ELISA of MMP-3 protein levels in conditioned media collected from PC3 and DU145 prostate cancer cells (**a**), paired benign/normal- and cancer-associated prostate fibroblasts of two patients (**b**) or HS27A bone stromal fibroblasts (**c**) treated with or without hydrogen peroxide and TGF-β1, either alone or in combination. The experiments were done at least three times in triplicates. Data are presented as the mean ± SD of triplicate determinations of one representative experiment. **Student’s *t*-test *p* < 0.001.

Cancer cells produce a pro-oxidant environment because of increased ROS, such as superoxide and hydrogen peroxide^24^. A previous study demonstrated that androgen-independent prostate cell lines, such as PC3 and DU145, produce more hydrogen peroxide than an androgen-dependent cell line, such as LNCaP^25^. The present study also found that higher levels of hydrogen peroxide were constitutively produced in primary cultured CAFs compared with match-paired BAFs (Supplemental Fig. s4c). To further validate the effect of hydrogen peroxide on the dysregulation of MMP-3 in both tumor epithelial and stromal components of prostate cancer, MMP-3 levels of prostate cancer cells, prostate stromal fibroblasts, and bone stromal cells were determined in the absence and presence of hydrogen peroxide. ELISA showed that hydrogen peroxide markedly increased MMP-3 from a basal level of 78.80 ± 1.06 to 147.80 ± 2.26 pg/ml in PC3 cells and from an undetectable level to 19.8 pg/ml in DU145 cells. By contrast, hydrogen peroxide treatment reduced MMP-3 in prostate stromal cells, both BAFs (404.30 ± 13.74 to 1.30 ± 0.04 pg/ml in BAF-1, and 760.70 ± 6.50 pg/ml to nondetectable in BAF-2), and CAFs (135.80 ± 3.00 to 9.80 ± 0.07 pg/ml in CAF-1, and 234.40 ± 1.30 pg/ml to nondetectable in CAF-2) (Fig. 4b). Moreover, a decrease in MMP-3 was observed in HS27A bone stromal fibroblasts after treatment with hydrogen peroxide (Fig. 4c). Unlike hydrogen peroxide, exogenous TGF-β1 alone had no effect on the expression of MMP-3 in both prostate cancer cell lines, and no consistent regulatory pattern in prostate stromal fibroblasts was observed. In addition, although additional TGF-β1 resulted in some enhancement of hydrogen peroxide-induced MMP-3 expression in PC3 cells, its effect was not observed in the other tested cell lines. Taken together, these results demonstrate that hydrogen peroxide is a key mediator regulating MMP-3 levels in both prostate cancer cells and environmental fibroblasts, but it acts in opposite manners in these two cellular components.

### Hydrogen peroxide affects the promoter activity of MMP-3 in stromal fibroblasts but not in prostate cancer cells

**Figure 5:**
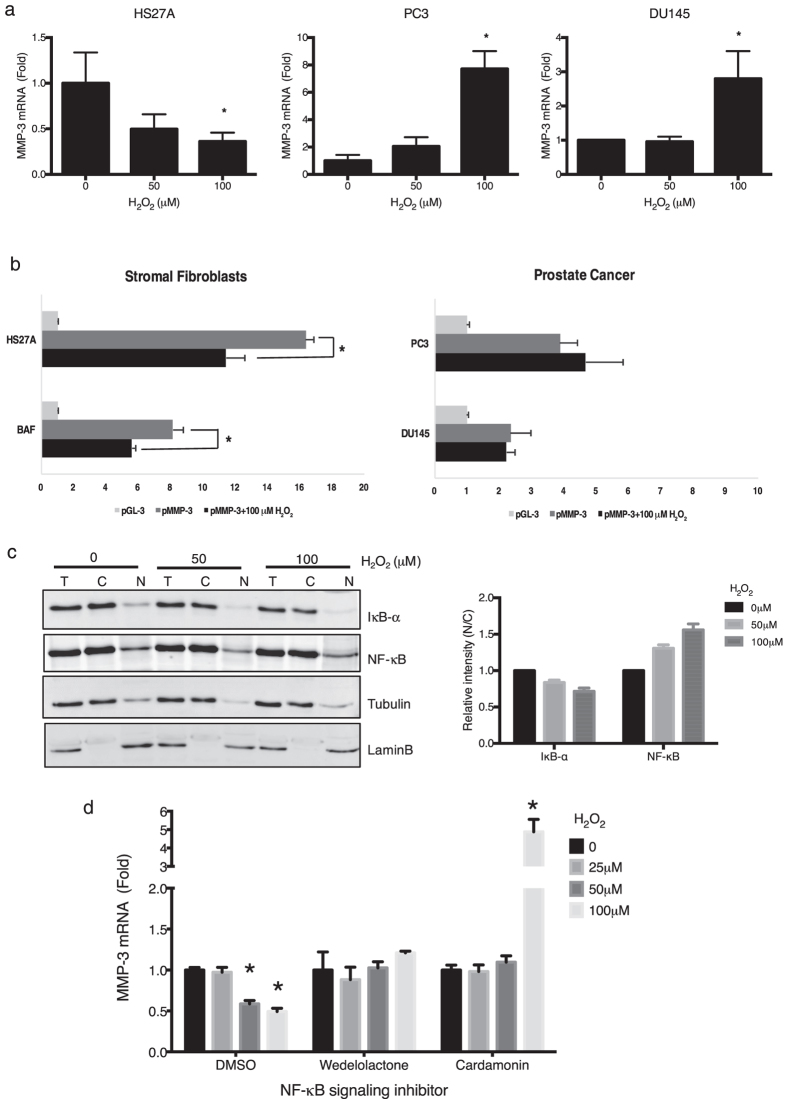
Effect of hydrogen peroxide on transcriptional activity of the MMP-3 promoter and nuclear factor (NF)-κB in stromal fibroblasts and prostate cancer cells. Comparison of endogenous MMP-3 mRNA levels (**a**), luciferase reporter activity of an MMP-3 promoter construct (pMMP-3) (**b**) and IκB-α and NF-κB/p65 protein levels (**c**) with or without hydrogen peroxide treatment for 48 h in stromal fibroblasts and prostate cancer cells through a real-time RT-PCR, luciferase assay and immunoblotting analysis, respectively. HS27A cells were pretreated with 10 μM NF-κB signaling pathway inhibitor (Wedelolactone and Cardamonin) or vehicle (DMSO) for 30 mins, followed by hydrogen peroxide treatment in the presence of corresponding inhibitor at 1 μM (**d**). The relative promoter activity of pMMP-3 was divided by the normalized activity of the empty vector (pGL3) and is expressed as a multiple of change over the control (**b**). Tubulin and lamin B were used as the loading controls of whole-cell lysate (T), the cytoplasmic fraction (C), and nuclear fraction (N), respectively (**c**). Band intensities were quantified and normalized relative to the quantity of their respective control bands and plotted as the multiple of change relative to the untreated group. Data are representative of three independent experiments and are shown as the mean ± SD. *Student’s *t*-test *p* < 0.05.

To further understand the possible mechanism underlying the bidirectional regulation of MMP-3 expression by hydrogen peroxide in a cell type-dependent manner, we first investigated whether hydrogen peroxide is involved in the transcriptional regulation of the *mmp-3* gene by determining the messenger (m)RNA expression level of MMP-3. Consistent with the ELISA data at the protein level, real-time RT-PCR analysis showed that *mmp-3* mRNA expression dose dependently decreased in HS27A stromal cells and increased in prostate cancer PC3 and DU145 cells following hydrogen peroxide treatment (Fig. 5a). Therefore, we explored the possible direct effect of hydrogen peroxide on the promoter activity of the *mmp-3* gene. DNA fragments located at −4283 to +19 bp upstream of the transcription start site of the *mmp-3* gene were cloned into a luciferase reporter vector to assess their transcriptional activities. A luciferase reporter assay demonstrated that hydrogen peroxide reduced the activity of 4-kb promoters in stromal cells, including bone stromal (HS27A) and prostate stromal (BAF) cells (Fig. 5b). However, although the mRNA level of *mmp-3* was upregulated by hydrogen peroxide in prostate cancer cells, we detected no significant differences in exogenous *mmp-3* promoter activities in PC3 prostate cancer cells after hydrogen peroxide treatment.

ROS have various inhibitory or stimulatory roles in NF-κB signaling^26^, a transcriptional factor that regulates MMP-3 expression^27, 28^. Therefore, we further explored the relationship between hydrogen peroxide-modulated MMP-3 transcription and the NF-κB pathway through Western blot analysis. We found that nuclear translocation of NF-κB p65 in HS-27A stromal cells increased concomitantly with a reduction in the level of IκB-α in a hydrogen peroxide dose-dependent manner (Fig. 5c). In addition, treatment of HS27A cells with NF-κB signaling pathway inhibitors targeting either NF-κB nuclear translocation (Cardamonin) or the upstream IKK-α and -β kinase activity (Wedelolactone) significantly attenuated the hydrogen peroxide-suppressed MMP-3 mRNA expression (Fig. 5d). These observations indicate that the hydrogen peroxide-mediated reduction in MMP-3 in stromal fibroblasts was, at least in part, possibly due to the suppression of promoter activities by NF-κB, whereas the induction of MMP-3 in prostate cancer cells by hydrogen peroxide may occur through other regulatory mechanisms, such as mRNA translation or stability.

### Hydrogen peroxide reverses THBS2-suppressed MMP-3 expression in prostate cancer cells through upregulation of miR-128

A growing body of evidence suggests that intracellular ROS production is an upstream event of miRNAs in response to cellular stress, which generates subsequent biological effects through the regulation of their direct target genes^29^. To determine whether the upregulation of MMP-3 in prostate cancer cells by hydrogen peroxide is linked to miRNA-mediated posttranscriptional regulation, we first focused on miR-888, miR-6800, and miR-371, which were predicted to be *mmp-3*-targeting miRNAs by two algorithms (miRNA.org and TargetScan). Real-time RT-PCR revealed that hydrogen peroxide caused no significant changes in the expression levels of these tested miRNAs in PC3 or DU145 cells. Because miRNAs are characterized as gene silencers that negatively regulate gene expression, the miRNA-targeted gene involved in hydrogen peroxide-induced MMP-3 upregulation is therefore most likely to be MMP-3 suppressors and not promoters. Thrombospodin 2 (THBS2) has an ability to modulate extracellular levels of MMPs^30^. A recent study demonstrated a suppressive role of THBS2 in MMP-3 expression to modulate prostate cancer metastasis^31^. With this evidence, we deduced a connection between THBS2 and hydrogen peroxide/miRNA-upregulated MMP-3 in prostate cancer cells.

**Figure 6:**
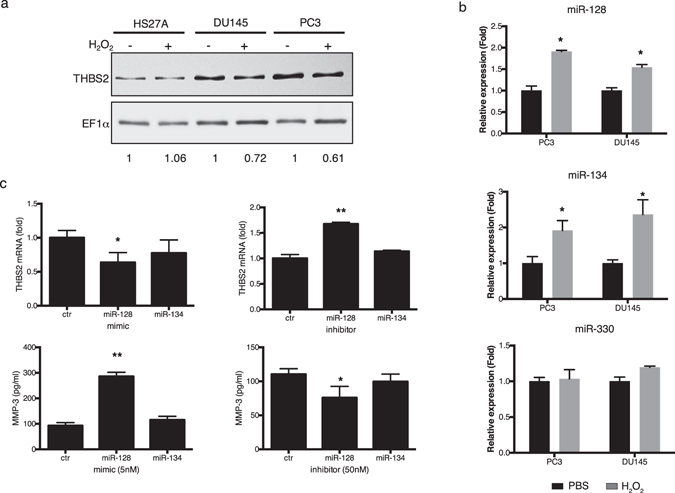
Involvement of thrombospondin-2 (THBS2) and microRNA (miR)-128 in hydrogen peroxide-upregulated matrix metalloproteinase-3 (MMP-3) in prostate cancer cells. Comparison of THBS2 (**a**) and miR-128, -134, and -330 (**b**) in PC3 and DU145 prostate cancer cells with or without 50 μM hydrogen peroxide treatment for 48 h through Western blot analysis (**a**) and a real-time RT-PCR (**b**), respectively. Real-time RT-PCR of THBS2 mRNA expression level and ELISA of MMP-3 levels in conditioned media of PC3 transfected with miRNA mimics or inhibitors (**c**). Data are representative of at least three independent experiments and are shown as the mean ± SD. *Student’s *t*-test *p ≤ 0.05; **p ≤ 0.001 compared with the control group.

THBS2 is a direct target of miR-128, -134, and -330^31^. We found that after hydrogen peroxide treatment, THBS2 protein decreased in DU145 and PC3 but not in stromal HS27A cells (Fig. 6a). This decreased expression of THBS2 was indeed correlated with the increased expression of miR-128 and -134 but not miR-330 (Fig. 6b). To determine whether the increase in miR-128 or -134 induced MMP-3 expression, we transfected an miR-128 or -134 mimic into PC3 cells. The miR-128 mimic significantly decreased THBS2 mRNA level more than 40% and increased MMP-3 protein expression by approximately three times of that in the nontargeting negative control mimic transfection, and the miR-134 mimic only slightly decreased THBS2 and increased the MMP-3 level (Fig. 6c). Conversely, transfection of an miR-128 inhibitor resulted in a significant increase in the expression of THBS2 and decrease in the MMP-3 level than that of the miR-134 inhibitor and the negative control miRNA inhibitor. Taken together, these results indicate that miR-128 plays a major role in hydrogen peroxide–induced MMP-3 expression in prostate cancer cells through the downregulation of THBS2.

## Discussion

The stromal microenvironment is a key player in the growth and development of cancer. Although numerous reports have elaborated the coevolution of prostate cancer cells with stroma in their genotypic and phenotypic characters, relatively few studies have delineated the contribution of CAFs to overall changes in gene and protein expressions in prostate cancer microenvironments. Herein, we demonstrated a trend of an increase in the MMP-3 level in the serum of prostate cancer patients with a pathological grade of the cancer. However, MMP-3 was upregulated in prostate cancer cells but downregulated in CAFs isolated from prostate stroma (primary culture) or derived from bone marrow stroma (3D coculture). In prostate cancer specimens, localization of MMP-3 staining was found primarily in tumor cells and much less in the surrounding stroma, similar to the observations in pancreatic, breast, and lung carcinomas^23, 32^, implying that MMP-3 expression is stage- and cell type-dependent during cancer progression. Physiologically, the stroma of normal tissues functions as a physical barrier against carcinogenesis. MMP-3 expression switching from stromal fibroblasts to cancer epithelial cells might be explained by the mechanisms regulating the stroma’s efforts to maintain tissue integrity and homeostasis during neoplastic transformation. Because normal stroma possesses inherent plasticity to respond rapidly to neoplastic situations and given the biological role of tumor microenvironment-derived factors in tumor growth or metastasis^33^, it is reasonable to speculate that stromal fibroblast-derived MMP-3 has a protective role in prostate cancer tumorigenesis; therefore, MMP-3 expression is decreased when the environment is converted into a pathological entity, where CAFs favor tumor progression. By contrast, cancer cells are remarkably plastic and capable of expressing genes, such as MMP-3, that mimic surrounding stromal cells, leading to the morphological and behavioral transition from epithelium to mesenchyme for their aggressive phenotypes^34^. While investigating the extracellular function of MMPs in ECM remodeling, we observed prominent nuclear immunostaining for MMP-3 and MMP-10 in the cancerous lesions of prostate cancer. Previous studies have identified putative nuclear localization signals and demonstrated the stromelysin proteolytic activity and transcriptional activity of nuclear MMP-3^35, 36^. These findings, in conjunction with our observation, suggests an undefined role of nuclear MMP-3 in the development of prostate cancer. In addition, the elevation of MMP-3 serum levels in cancer patients but the lack of a significant association with clinicopathological features, as demonstrated by the present and earlier studies^37–39^, implies that serum MMP-3 might be a useful diagnostic tumor marker albeit one that cannot be used to prognosticate.

ROS are essential development and physiological stimuli as well as major mediators of many disorders, including prostate cancer^12, 40^. Recent studies of cancer development have demonstrated that inflammation and hypoxic reactions are critical steps in cancer progression^41, 42^. Cancer epithelial cells produce large amounts of hydrogen peroxide and other ROS types during stress responses, not only to induce genetic mutations in cancer cells for a more-malignant phenotype^43^ but also to induce the Warburg effect in neighboring stromal fibroblasts, leading to a myofibroblast transition and providing the necessary energy-rich microenvironment to facilitate tumor growth and angiogenesis^44, 45^. In the present study, we demonstrated that hydrogen peroxide is one of the potential signaling mediators regulating gene expression changes in CAFs. The altered gene profile (Supplemental Fig. s3), such as increases in growth factors (CTGF), cell adhesion molecules (CDH2 and CDH6), inhibition of metalloproteinases (TIMP-3), and a reduction in MMPs (MMP-3), is involved in the induction of myofibroblast (trans)-differentiation and wound healing;^46–48^ therefore, CAFs can behave as activated myofibroblasts to drive invasive cancer growth and metastasis^49, 50^. Other downregulated genes, including SELENBP1 and IGFBP7, seen in CAFs have previously been reported to be tumor suppressors with proapoptotic and antiproliferative effects on prostate cancer cells^51–53^. Thus, this gene profiling of CAFs potentially explains how CAFs are particularly resistant to chemotherapy or radiotherapy and how they participate in tumor relapse. Future therapeutic strategies will require the targeting of both cancer cells and CAFs.

Another candidate regulator seen in the signaling pathway analysis of differentially expressed genes in CAFs is TGF-β, which has been well-documented to induce fibroblast-to-myofibroblast switching through increases in the intracellular levels of ROS^54, 55^. We demonstrated that ROS (hydrogen peroxide) were elevated in both prostate cancer cells and CAFs. However, unlike hydrogen peroxide, treating cancer cells or fibroblasts with TGF-β had no significant effect on MMP-3 expression (Fig. 4). A TGF-β–inhibitory element is present in the promoter of rat MMP-3^56^, but it has not been identified in the human *mmp-3* promoter, indicating that ROS affect MMP-3 gene expression through a different intracellular signaling pathway independent of TGF-β. TGF-β is a multifunctional cytokine that can act as a tumor suppressor in epithelial cancers through the inhibition of proliferation and the induction of apoptosis. A recent report demonstrated that exogenous TGF-β1 inhibits proliferation in DU145 prostate cancer cells but exerted no effect on proliferation in PC3^57^. This selective anticancer proliferation may explain, in part, why TGF-β treatment slightly reduced the concentration of MMP-3 in the CM of DU145 but not PC-3 regardless of the presence of hydrogen peroxide (Fig. 4a). Likewise, TGF-β induces fibroblast proliferation, which could increase the MMP-3 concentration in BAF-1 and CAF-1 cultures (Fig. 4b). Moreover, it is not surprising to find a suppressive effect of TGF-β on MMP-3 expression and an additive effect on hydrogen peroxide-suppressed MMP-3 in stromal cells because TGF-β was primarily identified as a putative mediator for differential gene expression in CAFs through IPA analysis (Figs s3b and s4a). Other mechanisms contributing to the differential response to TGF-β modulating MMP-3 expression among different prostate cancer and stromal cell lines remain to be elucidated.

Although no consensus NF-κB element has yet been identified in the human *mmp-3* promoter and the mechanism through which the NF-κB suppresses *mmp-3* gene expression in CAFs but not in prostate cancer epithelial cells is yet to be determined, our study identified a preliminary association between ROS-mediated *mmp-3* promoter suppression and NF-κB activation in a cell type-dependent manner. Previous studies have identified that a transcriptional factor zinc-binding protein (ZBP)-89 complexed with NF-κB bound to the *mmp-3* promoter at a repressor element is involved in the inhibition of the induction of MMP-3 transcription by interleukin-1^58, 59^, increasing the probability that NF-κB may contribute to ROS-mediated human MMP-3 suppression in CAFs through interaction with ZBP-89 or other transcriptional repressors. In contrast to CAFs, we demonstrated for the first time that ROS increased MMP-3 expression in prostate cancer cells by upregulating miR-128, which targets the MMP-3 suppressor THBS2. These results reveal a regulatory process that is the reverse of the previous finding that MMP-3 stimulates ROS production, leading to genomic instability and subsequent malignant transformation^15, 23^, further supporting the cross-talk between ROS and MMPs (Supplemental Fig. s5). Most miRNAs examined in our study either increased or did not change after hydrogen peroxide treatment in prostate cancer cells. Thus, inhibition of the miRNA biosynthesis enzyme Dicer 1 leading to a global decrease in miRNA maturation by ROS^60^, at least in the concentration range of 25–100 µM of hydrogen peroxide, might not be the case for ROS-regulated mir-128 expression shown in our study. Several miRNAs, such as miR-9, miR-21, miR-143, miR-146, and miR-224, have been validated to be directly transcriptionally regulated by NF-κB^29^. Although *mmp-3* promoter activity was not affected by hydrogen peroxide in prostate cancer cells, concomitant increases in the expressions of NF-κB and MMP-3 were observed, suggesting that NF-κB may have an indirect mechanism of upregulating MMP-3 expression by transcriptionally targeting its regulatory miR-128.

In summary, our results indicate that ROS play a crucial role in prostate cancer progression by modulating key elements of ECM homeostasis control. In the complex, ROS represent main factors in the interplay between prostate cancer cells and the stromal environment. Additional studies are necessary to better understand the molecular mechanisms controlling these processes by ROS.

## Methods

### Cell lines and cell culture

Human prostate cancer epithelial cell lines, LNCaP, C4-2, PC3, PC3M, and DU145, and a human bone stromal cell line HS27A (ATCC, Manassas, VA) that were used in our previous studies^61–63^ were maintained in T-medium (Invitrogen, Carlsbad, CA) with 5% fetal bovine serum (FBS; Invitrogen). Then, the 3D coculture of HS27A cells mixed with equal numbers of LNCaP or C4-2 cells or a monoculture was performed in an RCCS system (Synthecon, Houston, TX) following an established protocol^21^. Each condition was performed in duplicate vessels for three independent experiments. Previously established CAFs and BAFs, derived from cancerous and benign/normal regions of the prostate gland, respectively^21^, were confirmed as fibroblasts by positive vimentin but negative cytokeratin expression (Supplemental Fig. s6) and were maintained in PrEGM™ Prostate Epithelial Cell Growth Medium (Lonza, Walkersville, MD). All cells were cultured in a 37 °C incubator with 5% CO_2_.

### Western blot analysis

Cytoplasmic and nuclear proteins were extracted using a commercial kit (FIVEphoton Biochemicals, San Diego, CA) according to manufacturer instructions. Western blot analyses of cell lysates were performed as previously described^63^, except that the blots were probed using antibodies against IκB-α, NF-κB p65 (Cell Signaling, Beverly, MA), or thrombospondin 2 (THBS2; Boster Biological Technology, Pleasanton, CA). For the loading control, blots were probed with an anti-EF1α (R&D Systems, Minneapolis, MN), anti-tubulin, and anti-lamin B antibodies (GeneTex, Irvine, CA). Chemiluminescent signals were detected using an enhanced chemiluminescence (ECL) system (Advansta, Menlo Park, CA) and quantified using ImageJ software.

### Immunohistochemical (IHC) staining

A prostate adenocarcinoma tissue microarray was obtained from Super Bio Chips (CA4, Seoul, Korea), which contained 49 tissue cores representing samples from 40 cases of prostate cancer and nine matched normal adjacent tissues. After deparaffinization, rehydration, and antigen retrieval, the tissue sections were processed using the Novolink Polymer Detection System (Leica Microsystems, Newcastle Upon Tyne, UK) as previously described^62, 63^. Rabbit polyclonal antihuman MMP-3 (1:250 dilution, LifeSpan BioSciences, Seattle, WA) and rabbit polyclonal antihuman MMP-10 (1:400 dilution, LifeSpan BioSciences) antibodies were used for staining. Stained slides were scanned, and the images were digitized using the TissueFAXS system (TissueGnostics, Vienna, Austria) equipped with a 20× objective.

### ELISA of MMP-3 and -10

Cells were seeded in 6-well plates (2.5 × 10^5^ cells/well) with the respective culture medium. After the cells had attached to the bottom of the plate, the culture medium was washed with phosphate-buffered saline (PBS), and the medium was changed to RPMI 1640 (Hyclone, Logan, UT) without FBS for 48 h. CM was collected and filtered through a 0.2-µm filter. Experiments with human samples were reviewed and approved by the Joint Institutional Review Board of Taipei Medical University (TMU-JIRB 201312052). Serum from patients who were diagnosed with BPH and prostate cancer was obtained from the TMU bio-bank. All patients provided informed consent for the use of their blood samples and clinical data for research. Concentrations of MMP-3 and -10 in CM and patient serum were determined using the Quantikine® ELISA system (R&D Systems) following manufacturer recommendations. The optical density was determined at 450 nm, and readings at 570 nm were subtracted to correct for optical imperfections in the plate by using a Varioskan Flash Multimode Reader (Thermo Scientific, Waltham, MO).

### Animal study

All animal experiments were approved by and complied with the regulations of Taipei Medical University (LAC-2013-0109). Six-week-old male athymic nude mice BALB/cAnN.Cg-Foxn1nu/CrlNarl mice were obtained from the National Laboratory Animal Center (Taipei, Taiwan). Animals were kept under standard pathogen-free conditions and cared for according to the criteria outlined in the *National Academy of Sciences Guide for the Care and Use of Laboratory Animals*. C4-2 cells at 5 × 10^6^, either alone or mixed with an equal number of stromal cells in 100 μL of PBS, were subcutaneously injected into the flanks of the mice (*n* = 8 in each group); the experiments were independently conducted twice. Bidimensional tumor measurements were performed weekly by using calipers, and the tumor volume was calculated using the simplified formula for a rotational ellipsoid (L × W^2^ × 0.5236). Animals were euthanized using CO_2_ 8 weeks (end point) after cell inoculation.

### Promoter construction and luciferase reporter assay

A 4-kb fragment (−4283 to +19 bp) of the MMP-3 promoter construct (pMMP-3) was amplified from genomic DNA of PC3 cells using PCR and subsequently cloned into the vector pGL3-basic (Promega, Madison, WI) containing the coding region of the firefly luciferase gene. A vector pGL3-basic or pMMP-3 luciferase reporter plasmid was mixed with pCMV-βgal (galactosidase) at a 5:1 molar ratio and transfected into cells with Lipofectamine 2000 transfection reagent (Invitrogen). After 6 h of transfection, the cells were treated with 50-µM hydrogen peroxide for 48 h and subjected to luciferase and β-gal activity measurements using the Luciferase Assay System and β-Galactosidase Enzyme Assay system (Promega) according to manufacturer instructions. The relative luciferase activity was calculated by dividing the firefly luciferase relative light units (RLU) by the corresponding value for β-gal activity. Three independent experiments were performed in triplicate.

### Reverse-transcription (RT)-PCR

Total RNA was extracted with the Trizol reagent (Invitrogen) and subjected to first-strand complimentary (c)DNA synthesis using random primers and Moloney murine leukemia virus reverse transcriptase (Invitrogen) for messenger (m)RNAs or miScript Reverse Transcription Kit for microRNAs (miRNAs). After cDNA synthesis, a real-time PCR was performed using the LightCycler 480 with the Light Cycler TaqMan master kit combined with the Universal ProbeLibrary probe (Roche, Indianapolis, IN) for MMP-3 and THBS2 detection, as previously described^62^. Mature miRNAs were amplified from cDNA templates using the miScript SYBR Green PCR Kit and miScript Primer Assay according to manufacturer instructions (Qiagen, Hilden, Germany). HSPCB and RNU6B were respectively used as the reference genes for mRNAs and miRNAs. Specific gene expression levels were normalized to the reference and to the mean expression of normal samples using the 2^−ΔΔCt^ method.

### Recombinant proteins and chemicals

Hydrogen peroxide solution (30%) was purchased from Sigma-Aldrich (St. Louis, MO). Recombinant human TGF-β was obtained from R&D Systems. Specific microRNA mimics and inhibitors for miR-128 (MSY0000424 and MIN0000424), miR-134(MSY0000447 and MIN0000447), and miR-330 (MSY0000751 and MIN0000751) were purchased from Qiagen. IKK inhibitor II (Wedelolactone) and NF-κB inhibitor (Cardamonin) was obtained from Millipore Corporation (Billerica, MA) and Tocris Bioscience (Ellisville, MO), respectively.

### Statistical analysis

All data from the *in vitro* studies are presented as mean ± standard deviation (SD). Differences between groups were analyzed using a two-tailed Student’s *t*-test or as indicated in the figure legend. *p* < 0.05 was considered significant.

## Electronic supplementary material

Supplementary information
