# Supplementary material for: Reactive oxygen species–mediated switching expression of MMP-3 in stromal fibroblasts and cancer cells during prostate cancer progression
Source: Sci Rep. 2017 Aug 22;7:9065. doi: 10.1038/s41598-017-08835-9 (PMC5567216; doi:10.1038/s41598-017-08835-9)
Supplement: Supplementary file 1 — Supplementary information [file 41598_2017_8835_MOESM1_ESM.pdf]

# Supplementary data

## Reactive oxygen species–mediated switching expression of MMP-3 in stromal fibroblasts and cancer cells during prostate cancer progression

Chia-Ling Hsieh[1,+], Che-Ming Liu[1,2,+], Hsin-An Chen[3,4,5], Shun-Tai Yang[3,5,6], Katsumi Shigemura[7,8], Koichi Kitagawa[7,8], Fukashi Yamamichi[9], Masato Fujisawa[7,8], Yun-Ru Liu[10], Wei-Hua Lee[11], Kuan-Chou Chen[5,12,13], Chia-Ning Shen[2,14], Cheng-Chieh Lin[2], Leland W. K. Chung[15], Shian-Ying Sung[1,16,*]

[1]The Ph.D. Program for Translational Medicine, College of Medical Science and Technology, Taipei Medical University, Taipei, Taiwan, [2]The Ph.D. Program for Cancer Biology and Drug Discovery, China Medical University and Academia Sinica, Taichung, Taiwan, [3]Department of Surgery, School of Medicine, College of Medicine, Taipei Medical University, Taipei, Taiwan, [4]Division of General Surgery, Department of Surgery, Shuang Ho Hospital, Taipei Medical University, New Taipei City, Taiwan, [5]Graduate Institute of Clinical Medicine, College of Medicine, Taipei Medical University, Taipei, Taiwan, [6]Department of Neurosurgery, Shuang Ho Hospital, Taipei Medical University Taiwan, [7]Department of Urology, Kobe University Hospital, Kobe, Japan, [8]Department of International Health, Kobe University Graduate School of Health Science, Kobe, Japan, [9]Department of Urology, Hyogo Prefectural Amagasaki Hospital, Amagasaki, Hyogo Prefecture 660-0828, Japan, [10]Joint Biobank, Office of Human Research, Taipei Medical University, Taipei, Taiwan, [11]Department of Pathology, Shuang Ho Hospital, Taipei Medical University, New Taipei City, Taiwan, [12]Department of Urology, Shuang Ho Hospital, Taipei Medical University, New Taipei City, Taiwan, [13]Department of Urology, College of Medicine, Taipei Medical University, Taipei, Taiwan, [14]Genomic Research Center, Academia Sinica, Taipei, Taiwan, [15]Uro-oncology Research Program, Samuel Oschin Comprehensive Cancer Institute, Cedars-Sinai Medical Center, Los Angeles, CA 90048, USA, [16]Joint Clinical Research Center, Office of Human Research, Taipei Medical University, Taipei, Taiwan

*Corresponding author
[+]These authors contributed equally to this work.

## Supplementary Table

Table S: List of primers and probes used in quantitative PCR

| Gene | Primer sequences* | UPL† |
|---|---|---|
| MMP-3 | F: CAAAACATATTTCTTTGTAGAGGACAA<br>R: TTCAGCTATTTGCTTGGGAAA | No.36 |
| MMP-10 | F: CAAAAGAGGAGGACTCCAACA<br>R: TTCACATCCTTTTCGAGGTTG | No.61 |
| HSPCB | F: AGCCTACGTTGCACTATTACG<br>R: GAAAGGCAAAAGTCTCCACCT | No.55 |
| THBS2 | F: GTGCAGGAGCGTCAGATGT<br>R: GGGTTGGATAAACAGCCATC | No.17 |

* F: forward primer; R: reverse primer
† Universal Probe Library (Roche Applied Science)

**Supplementary Figures**

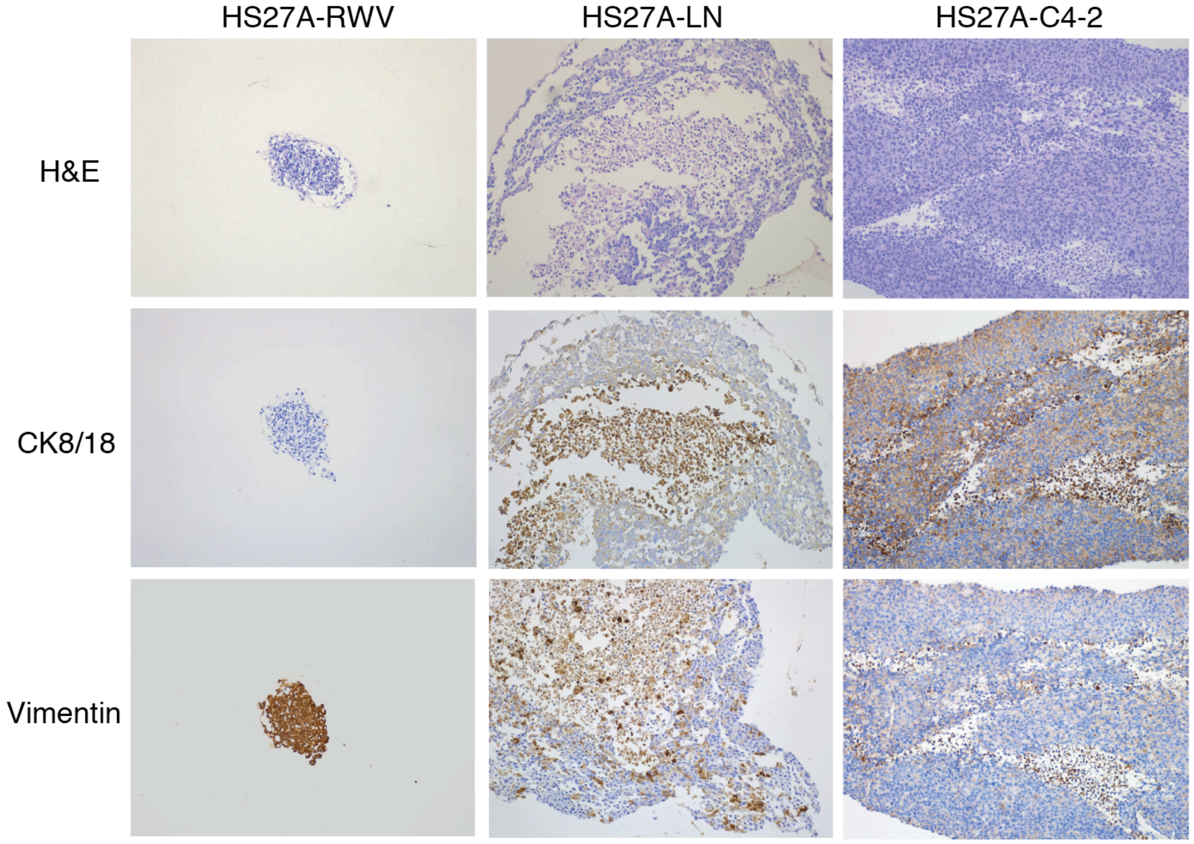


Figure s1. Detection of mixed cellular components in the 3-D cultured prostate tumoroids by immunohistochemical analysis. Cellular aggregates harvested from the HS-27A monoculture (HS27A-RWV) and the coculture of HS-27A mixed with prostate cancer cells LNCaP (HS27A-LNCaP) or C4-2 (HS27A-C4-2) were embedded in agarose and subjected to tissue section. The cell morphology was assessed by H&E staining, and the presence of prostate cancer cells (luminal epithelial cells) and HS27A (fibroblasts) was characterized using immunohistochemical staining with antibody against human cytokeratin 8 and 18 (CK 8/18) and vimentin, respectively. The original magnification was ×100.

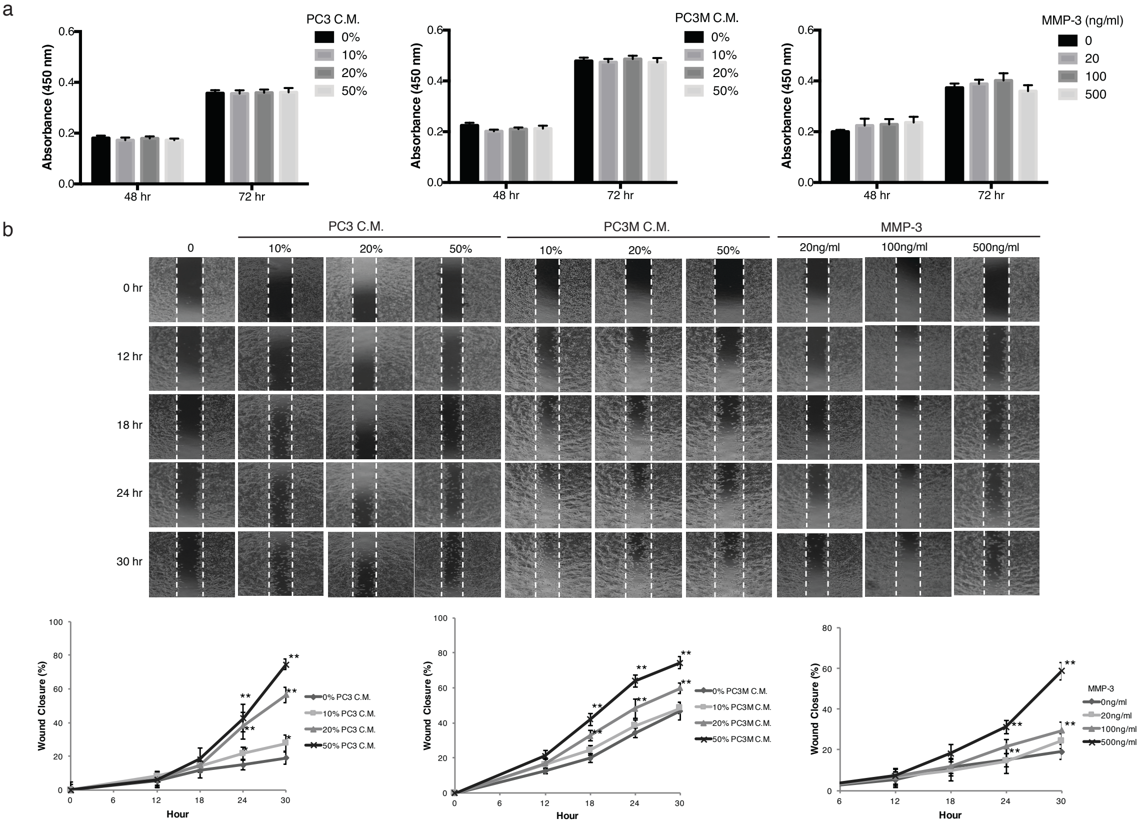


Figure s2. Effect of conditioned medium and MMP-3 on prostate cancer cell growth and migration. Serum-free conditioned medium (C.M.) harvested from highly metastatic prostate cancer PC3 and PC3M cells were prepared as 10%, 20% and 50% of working solution in PRMI medium supplemented with 5% FBS. The low aggressive prostate cancer C4-2 cells were treated with C.M. or recombinant human MMP-3 for growth and migration assessment using WST-1 cell proliferation assay (a) and wound healing assay (b), respectively. The relative cell number was assessed at 48 h and 72 h after treatment and presented as absorbance at 450 nm. Error bars indicate SD of eight replicates measurements (a). Wound healing assay was performed on confluent cells grown overnight with wound healing culture inserts. The cells were subsequently treated with C.M. or MMP-3 when the inserts were removed. The residual gap area was imaged (original magnification x40) at the indicated time point and measured by ImageJ software (b). Representative images for each condition were shown. The dashed lines indicate the initial edge of the gap. The quantitative data was presented as mean ± SD of one experiment performed in triplicate wells and are representative of three independent experiments. *$p < 0.05$, ** $p < 0.001$ versus the same time points of blank medium (0% or 0ng/ml).

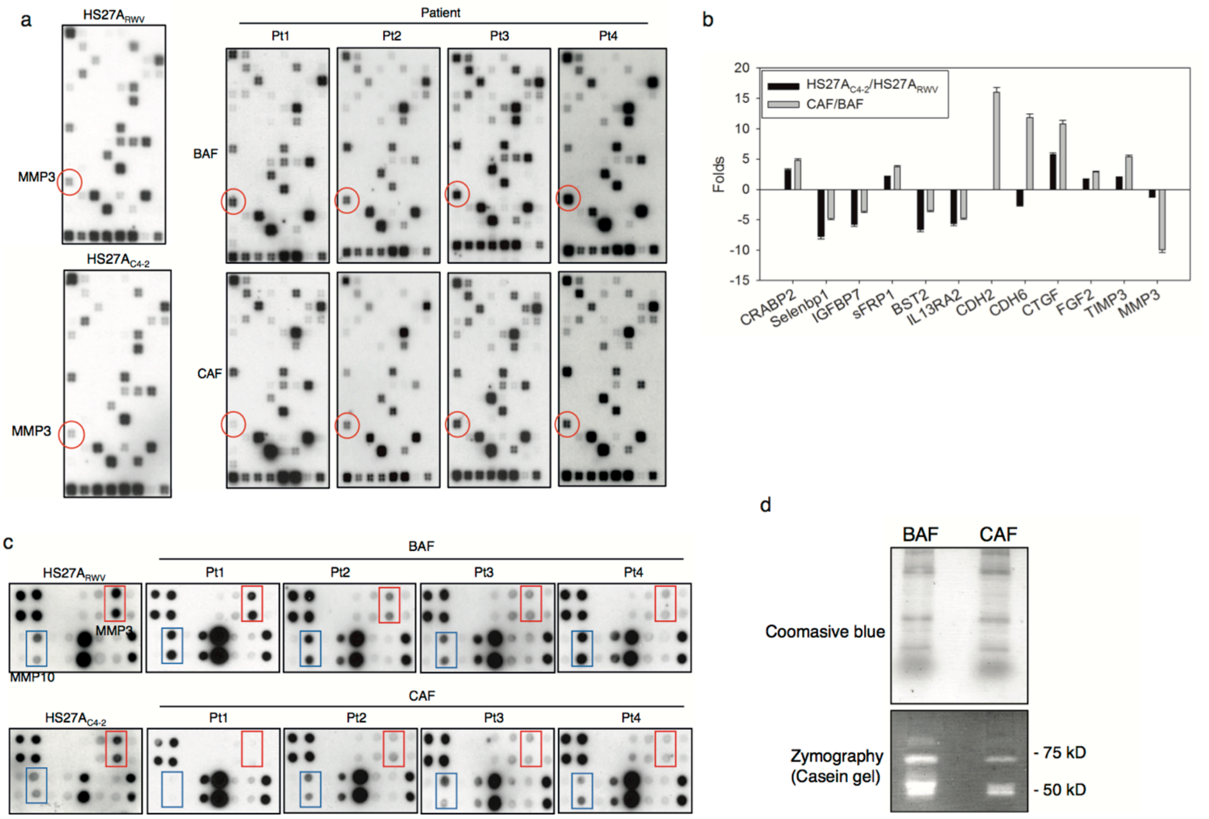


Figure s3. Detection of upregulated matrix metalloproteinase (MMP)-3 and -10 expressions in cancer-associated stromal fibroblasts by array assays. A pathway-focused mRNA array (SuperArray Bioscience Corporation, Frederick, MD) (a) and antibody array (RayBiotech, Norcross, GA) (c) revealed the differential expressions of MMP-3 and/or MMP-10 between normal and cancer-associated stromal cells in the set of HS27A (HS27A$_{RWV}$ *vs*. HS27A$_{C4-2}$) and four paired patient-derived cell lines (benign-associated (BAFs) *vs*. cancer-associated fibroblasts (CAFs). The location of MMP-3 and -10 detection on the membrane was indicated. Validation of the alteration of gene expression found in the mRNA array by a real-time PCR (b). Casein zymogram photomicrograph of conditioned media of BAF and CAF revealed a significantly decreased ability of substrate degradation, with Coomassie blue gel staining as a loading reference (d).

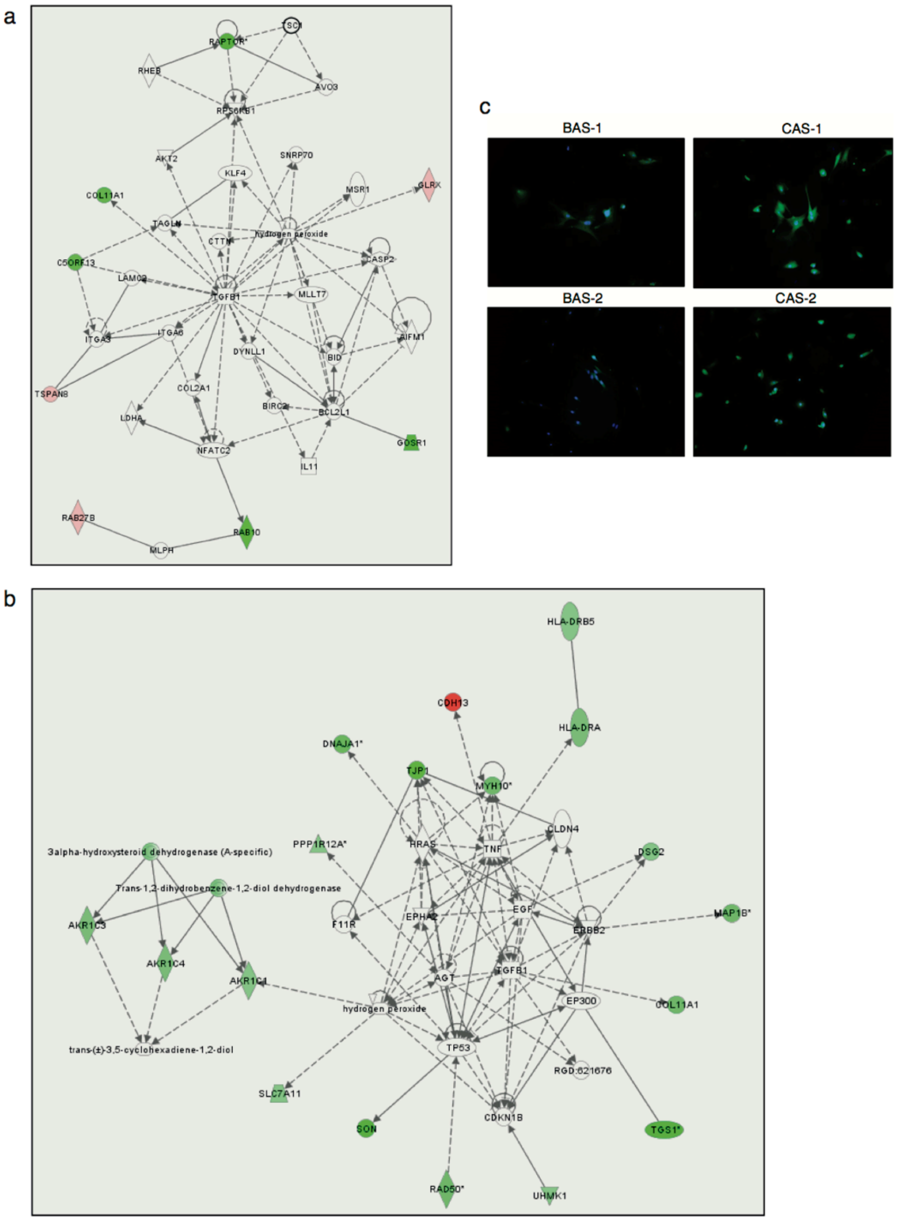


Figure s4. Network representation of differential gene expressions between normal and cancer-associated stromal cells. A comprehensive gene network that merged with the related signaling pathway in HS27A$_{C4\text{-}2}$ (a) and cancer-associated fibroblasts (CAFs) (b) compared to their corresponding normal stromal control was generated using Ingenuity Pathway Analysis software. Downregulated genes are shown in red, and upregulated genes in green. Transforming growth factor (TGF)-β1 and hydrogen peroxide were shared in common in both cell sets as the central mediator. Comparison of intracellular ROS ($H_2O_2$) levels between paired benign-associated fibroblasts (BAFs) and CAFs derived from two representative patients using 2',7'-dichlorofluorescein diacetate (DCF-DA) staining (c). The original magnification was ×100.

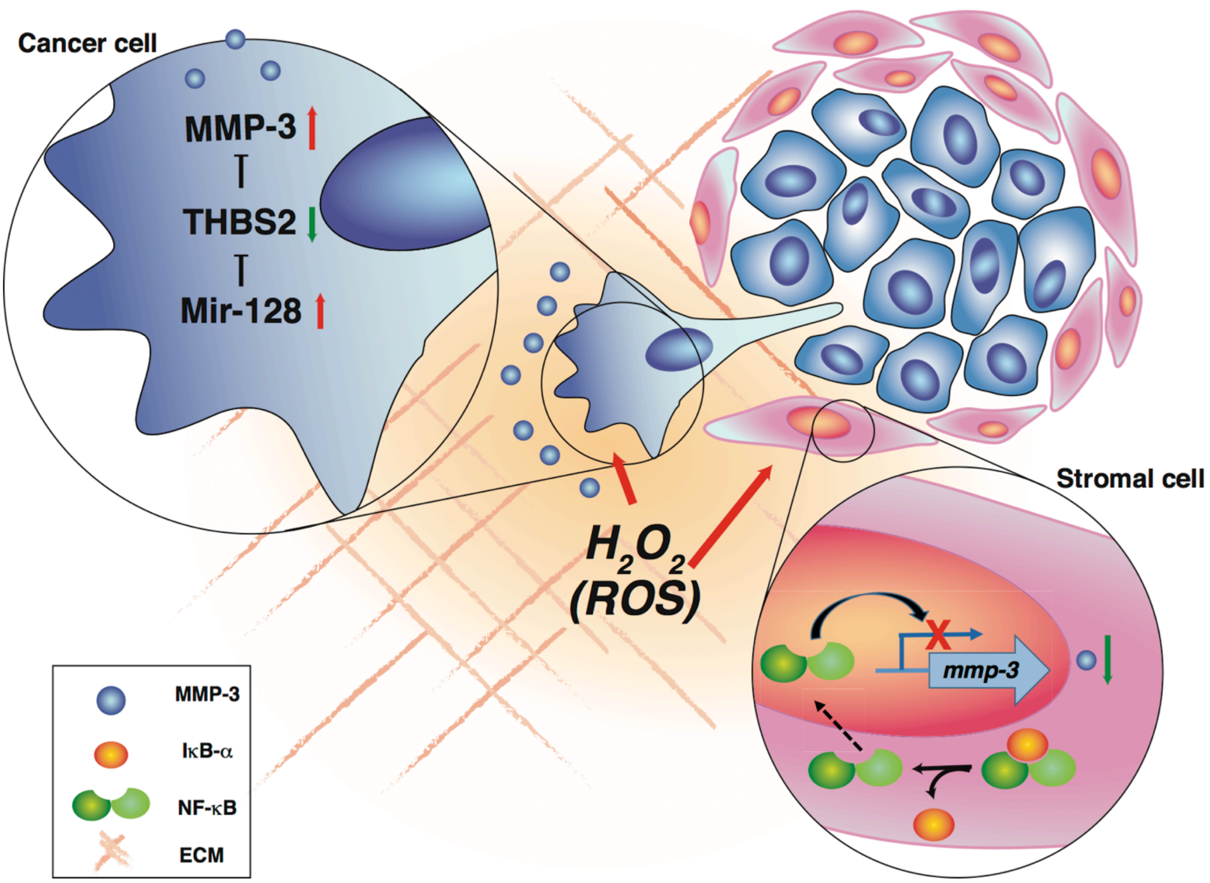


Figure s5. Scheme illustrating the pathways of hydrogen peroxide in switching expression of MMP-3 in stromal fibroblasts and prostate cancer cells.

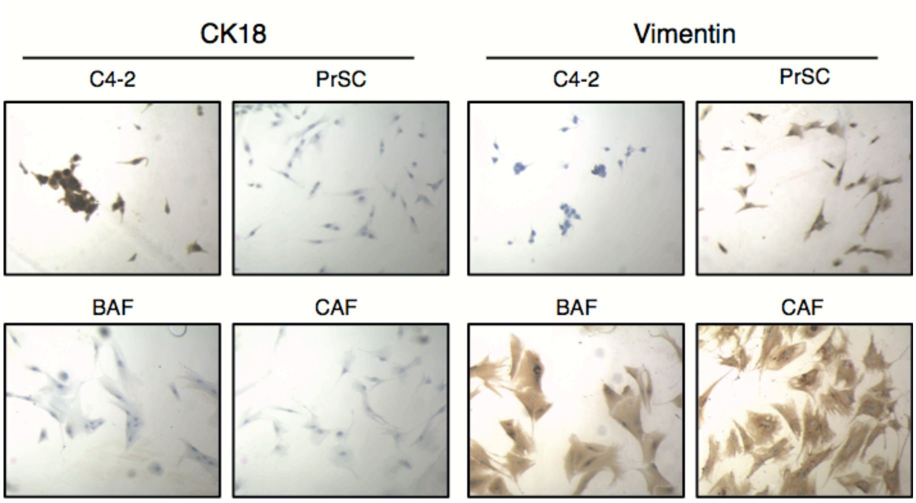


Figure s6. Characterization of stromal fibroblast origin of patient-derived prostate fibroblasts. Cell staining was negative for CK18 and positive for vimentin in benign/normal-associated (BAFs) and cancer-associated fibroblasts (CAFs) derived from prostate cancer patients. C4-2 prostate cancer cells and a commercialized prostate stromal cell line (PrSC) were respectively used as epithelial and stromal cell controls.

**Full length blots in Figure 5c**

37

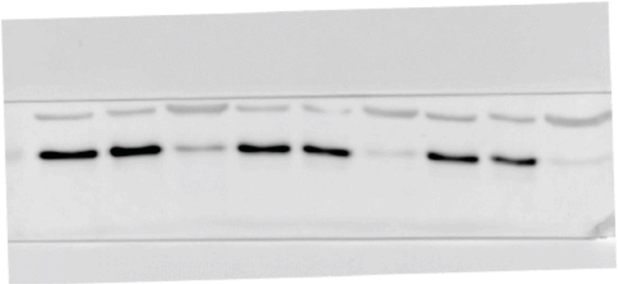

IkB-a (36kD)

75

50

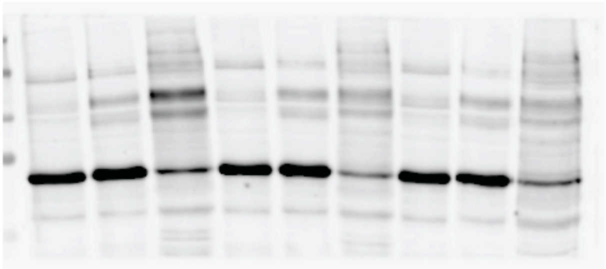

NF-kB (65kD)

75

50

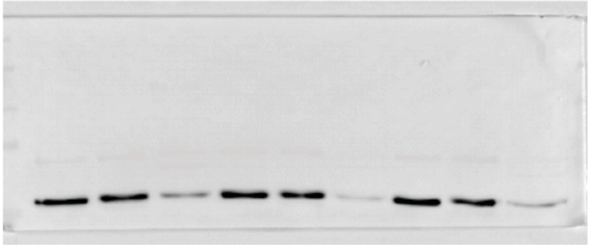

Tubulin (55kD)

75

50

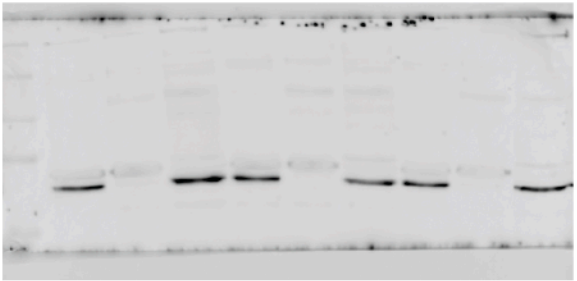

LaminB2 (68kD)
